# The transmembrane proteins (TMEM) and their role in cell proliferation, migration, invasion, and epithelial-mesenchymal transition in cancer

**DOI:** 10.3389/fonc.2023.1244740

**Published:** 2023-10-23

**Authors:** Gloria Angelina Herrera-Quiterio, Sergio Encarnación-Guevara

**Affiliations:** Laboratorio de Proteómica, Centro de Ciencias Genómicas, Universidad Nacional Autónoma de México, Cuernavaca, Morelos, Mexico

**Keywords:** TMEM proteins, cancer, proliferation, invasion, migration, chemotherapy

## Abstract

Transmembrane proteins (TMEM) are located in the different biological membranes of the cell and have at least one passage through these cellular compartments. TMEM proteins carry out a wide variety of functions necessary to maintain cell homeostasis TMEM165 participates in glycosylation protein, TMEM88 in the development of cardiomyocytes, TMEM45A in epidermal keratinization, and TMEM74 regulating autophagy. However, for many TMEM proteins, their physiological function remains unknown. The role of these proteins is being recently investigated in cancer since transcriptomic and proteomic studies have revealed that exits differential expression of TMEM proteins in different neoplasms concerning cancer-free tissues. Among the cellular processes in which TMEM proteins have been involved in cancer are the promotion or suppression of cell proliferation, epithelial-mesenchymal transition, invasion, migration, intravasation/extravasation, metastasis, modulation of the immune response, and response to antineoplastic drugs. Inclusive data suggests that the participation of TMEM proteins in these cellular events could be carried out through involvement in different cell signaling pathways. However, the exact mechanisms not clear. This review shows a description of the involvement of TMEM proteins that promote or decrease cell proliferation, migration, and invasion in cancer cells, describes those TMEM proteins for which both a tumor suppressor and a tumor promoter role have been identified, depending on the type of cancer in which the protein is expressed. As well as some TMEM proteins involved in chemoresistance. A better characterization of these proteins is required to improve the understanding of the tumors in which their expression and function are altered; in addition to improving the understanding of the role of these proteins in cancer will show those TMEM proteins be potential candidates as biomarkers of response to chemotherapy or prognostic biomarkers or as potential therapeutic targets in cancer.

## Introduction

**Figure 1:**
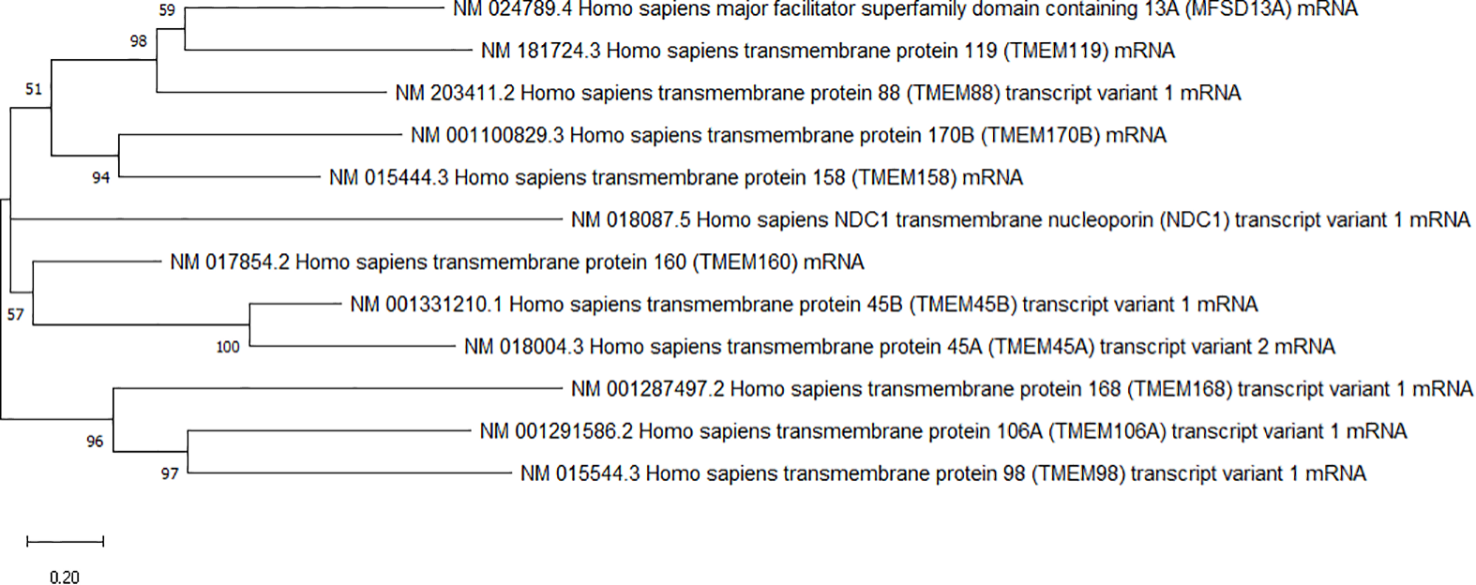
Phyllognetic tree of TMEM proteins. The tree was built using the maximum likelihood method using transcripts of de genes and the Mega 10.2.5 program.

Transmembrane proteins are present in all cell membranes. These kinds of proteins have at least one passage through the phospholipid bilayer and possess transmembrane, intra, and extracellular domains (1). The transmembrane domain fields comprise alpha helices or multiple beta sheets (2). Transmembrane proteins have a wide variety of functions in the cell that include the transport of molecules across membranes such as the aquaporin family that allow the passage of water and a wide variety of solutes and gases (3, 4), enzymatic activity (5), receptors for hormones (6), neurotransmitters (6, 7) lipoproteins (8) and an extensive number of ligands (7), allow the cell-cell communication (9) and with the extracellular matrix (9). It is estimated that humans have around 5,500 transmembrane proteins (10). Within these proteins, there is a group of transmembrane proteins classified as a family of TMEM proteins whose physiological function and structural characteristics are not fully understood, and which are phylogenetically related (**Figure 1**). The main reason they are grouped in this family is because they are located in the membrane and have not identified biological functions. Consequently, they are identified as transmembrane proteins (TMEM) plus a number as identifier (10).

TMEM proteins are located in different biological membranes. For example, TMEM180 is in the plasma membrane (11), TMEM165 is in the late Golgi (trans) (12), and endoplasmic reticulum (ER) (13), TMEM74 and TMEM9 in the lysosome membrane (14–16), TMEM106A (17) and TMEM160 in the mitochondrial membrane (18, 19), and TMEM48 in the nuclear membrane (20). For some TMEM proteins, their localization has been demonstrated experimentally for others, it has been predicted by bioinformatics tools (**Table 1**). When a TMEM protein is functionally characterized, it can be located in a new family of proteins according to its function and even given a new name; as an example, TMEM16A is a Ca2^+^ activated chlorine channel and was renamed anoctamin 1 (86), similarly TMEM206 which is a proton-activated chlorine channel and was renamed PAC (87).

**Table 1:** Summary of the main characteristics of TMEM proteins: name, transmembrane domains, chromosomal location, physiological function and role in cancer.

Protein TMEM	Localization	Domains	Chromosome	Physiological function	Cancer		Oncogene/ suppressor tumor	Reference
					Type of cancer	Processes involved in cancer		
TMEM45A	Endoplasmic reticulum and Golgi apparatus membrane	7	3	Epidermal keratinization	Ovarian cancer, Glioblastoma	Proliferation, invasion, and migration	Oncogene	(21–24; 13, 25–29)
					Colorectal cancer, cervical cancer	Proliferation, Invasion, and migration and EMT		
					Colorectal cancer	Chemorresistance		
TMEM45B	Endoplasmic reticulum membrane*, trans-Golgi, endosomes and lysosomes.	7*	11	Plays a role in innate immunity	Lung cancer, prostate cancer	Cell proliferation, metastasis	Oncogene	(13, 30–38)
					Pancreatic cancer	Cell proliferation, invasion, migration, apoptosis		
					Melanoma	Biomarker of progression		
					Gastric cancer, osteosarcoma	Cell proliferation, invasion, and migration		
TMEM119	Plasmatic membrane	1*	8	Bone formation and osteoclast differentiation	Gastric cancer, ovarian cancer, osteosarcoma	Proliferation	Oncogene	(39–47)
					Osteosarcome, gastric cancer, ovarian cancer	Migration, invasion		
					Ovarian cancer	EMT		
					Osteosarcome, Gastric cancer	Apoptosis		
					Breast cancer	Pluripotency		
TMEM48/ NCD1	Nuclear membrane	6	1	Nucleoporin participates in the assembly of the nuclear pore complex	Lung cancer, cervical cancer	Proliferation, adhesion, migration and	Oncogene	(48, 49)
					Lung cancer	Apoptosis		
TMEM168	Membrane	11*	7	NK	Glioblastoma	Cell cycle, proliferation, apoptosis.	Oncogene	(50–52)
TMEM98	Plasma membrane, endoplasmic reticulum membrane, and nuclear membrane	1	17	Th1 lymphocyte differentiation and inhibition of the Wnt / β-catenin pathway	Head and neck squamous cell carcinoma, gastric cancer, ovarian cancer	Proliferation, migration	Oncogene / suppressor tumor	(53–57)
					Gastric cancer, ovarian cancer	Invasion		
					Ovarian cancer	Apoptosis		
TMEM106A	Plasmatic membrane	1	17	Activation and polarization of M1 macrophages	Gastric cancer, renal cancer, lung cancer	Proliferation	Suppressor tumor	(17, 58–60)
					Renal cancer	Migration		
					Lung cancer	EMT		
					Gastric cancer, lung cancer, renal cancer	Apoptosis		
TMEM170B	Plasmatic membrane	3*	6	NK	Breast cancer	Cell proliferation	Suppressor tumor	(50, 61, 62)
					Pancreatic cancer	Invasion and migration		
					Pancreatic cancer	Infiltration immune cells		
TMEM158	Plasmatic membrane	2*	3	Neurotrophic peptide receptor derived from brain lesions	Lung cancer, colorectal cancer	Chemorresistance	Oncogene / suppressor tumor	(50, 63–71)
					Pancreatic cancer, breast cancer, glioblastoma	EMT		
					Ovarian cancer, colorectal cancer, glioblastoma	Proliferation, invasion, adhesion, migration		
					Prostate cancer	Infiltration immune cells		
					Laryngeal cancer, colorectal cancer	Apoptosis		
TMEM88	Plasmatic membrane and cytosolic	2	17	Negative regulation of the Wnt/β-catenin pathway, differentiation of cardiomyocytes	Lung cancer, thyroid cancer, hepatocellular carcinoma, ovarian cancer, colorectal cancer, glioma	Cell proliferation, invasion, migration.	Oncogene / suppressor tumor	(72–82)
TMEM180	Plasmatic membrane	12	10	It is proposed to be a cation symporter	Colorectal cancer	Nitric oxide (NO) synthesis system and glutamine and arginine metabolism	Oncogene / suppressor tumor	(11, 83–85)
					Colorectal cancer, pancreatic cancer	Cell proliferation		
TMEM160	Mitochondrial inner membrane	3*	19	It suppresses the production of reactive oxygen species and stabilizes mitochondrial proteins.	KN	Differential expression has been reported in different types of cancer, although its role in cancer has not been identified.	KN	(18, 19, 50)

Different and diverse functions have been identified for some TMEMs proteins: TMEM165 participates in protein glycosylation (12), TMEM74 regulates autophagy (16), TMEM45A contributes to epidermal morphogenesis (24), TMEM88 is essential for the development of cardiac cells (79). On the other hand, transcriptomic and bioinformatic studies have shown that there is a differential expression of TMEM proteins in different neoplasms concerning cancer-free tissues (24, 31, 61, 88–90). This has sparked interest in studying the role of TMEM proteins play in tumor cells.

Several reports have shown that some TMEM proteins have a specific role as oncogenes by promoting different processes in tumor cells as proliferation cell, migration, intravasation/extravasation, metastasis and modulation of the immune response (25, 91). For example, in glioblastoma cells TMEM45A promoting cell proliferation, migration, and invasion (27) and TMEM98 in gastric cancer (54), among others. Some TMEM proteins have also been reported to favor epithelial-mesenchymal transition (EMT), such as TMEM45A in colorectal cancer (28). While other TMEM proteins are associated with a tumor suppressor function, for example, TMEM106A favors the decrease in cell proliferation and migration in kidney cancer and lung cancer (59, 60) even though it has been reported that some TMEM proteins can exert both functions in different types of cancer, such is the case of TMEM180 that promotes cell proliferation in colorectal carcinoma (11), while in pancreatic cancer act as a tumor suppressor gene (92).

Reports on the roles of TMEM proteins in health and disease reported in the last decades have made the TMEM family of proteins a target of great research interest elucidating promises to identify potential therapeutic targets and biomarkers in cancer. This review describes the principal cellular processes in which the TMEM protein family has been implicated in cancer. For some TMEM proteins, the mechanism or signaling pathways in which they are involved in tumor cells are becoming increasingly clear. At the same time, for other members of the TMEM family, unfortunately, this has not happened. Therefore, understanding the role of TMEM proteins in tumor cells will improve understanding of cancer and thus identify potential biomarker candidates in this pathology.

## TMEM proteins promote cell proliferation, migration, and invasion in cancer

**Figure 2:**
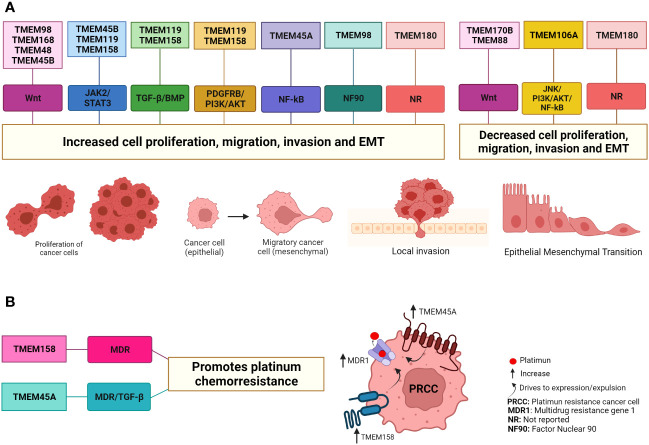
TMEM protein involved in cancer through different signaling pathways. **(A)** The TMEM98, TMEM168, TMEM48, TMEM45B, TMEM119 and TMEM45A proteins have been suggested as oncogenes in some types of cancer due to their role in increasing cell proliferation, migration, invasion and EMT. On the contrary, the proteins TMEM106A, TMEM170B and TMEM88 in some types of cancer are suggested as tumor suppressors because they decrease cell proliferation, migration, invasion and EMT. For some of these TMEM proteins it is a little clearer how they act through the signaling pathways while for other TMEM proteins it is not since the end effectors of each signaling pathway have been evaluated. In the case of the TMEM180 protein, it has been reported that it favors the increase and/or decrease of these cellular processes depending on the type of cancer, but it has not been reported through which signaling pathways it exerts this effect. **(B)** The TMEM158 and TMEM45 proteins favor platinum chemoresistance through the increase expression proteins associated with MDR and the signal pathway TGF-β. Created with Biorender.com.

Sustained proliferation is one of the main characteristics of tumor cells and is the result of cellular alteration at different levels (93, 94). This means that many proteins are involved in these cellular processes. In the last decade, a large number of investigations have reported that the levels of different TMEM proteins are positively regulated in cancer and that these proteins play a role in proliferation, migration, invasion, EMT, and metastasis and since they are located in the plasma membrane or organelle membrane and have a different number of transmembrane domains, their role in these cellular processes has been observed through different signaling pathways (**Table 1** and **Figure 2**).

In this way, TMEM45A, it is located in the Golgi apparatus (22, 24), also it is predicted to localize to the ER membrane and identified seven putative transmembrane domains (13, 91). This protein is expressed at high levels in the skin and participates in epidermal keratinization (24). The TMEM45A gene is positively regulated under hypoxic conditions by the hypoxia-inducible factor (HIF1α) (89). In ovarian cancer, it was reported that TMEM45A knockdown decrease cell proliferation, adhesion, and invasion. It was reported that in ovarian cancer cells, TMEM45A promotes cell proliferation by favoring the G1/S cell cycle transition. In addition, an association between TMEM45A and the regulation of TGF-β in ovarian cancer is suggested because the knockdown of TMEM45A significantly decreased the levels of the TGF-β1, TGF-β2, RhoA, and ROCK2 proteins that participate in the signaling pathway of TGF-β signaling (23).

In chemoresistant colorectal cancer cell lines, the knockdown of TMEM45 induced apoptosis and was correlated with a significant decrease in cell migration, invasion, EMT, and this last is associated with the TGF β/Smad signaling pathway (28). Zhang et al., reported high levels of TMEM45A mRNA in glioblastoma tissues. These were associated with reduced overall survival in patients. The knockdown of TMEM45A expression by CRISPR/Cas9 in glioblastoma cell lines A172 and U251 significantly decreased cell proliferation, migration, and invasion. At the same time, stable overexpression of TMEM45A reversed the silencing effect. Furthermore, the expression of nuclear factor kappa-B (NF-κB) as a gene potentially associated with TMEM45A was analyzed, and a positive correlation is shown (27). NF-κB is a transcription factor that regulates the expression of a large number of genes involved in cellular processes such as cell proliferation, differentiation. In glioblastoma, its activation has been observed in an aberrant constitutive manner and has been implicated in processes such as cell growth promotion and EMT, to name a few (95). In glioblastoma cells the expression of TMEM45A favored the expression of NF-κB. On the other hand, the silencing of NF-κB counteracted the proliferation of the cells that overexpress TMEM45A, these data suggest that in glioblastoma cells, TMEM45A could have an oncogenic role through regulation of NF-κB (27).

TMEM45A expression has also been reported to be upregulated in CaSki (+HPV 16), SiHa (+HPV 16), and HeLa (+HPV 18) cervical cancer cells, compared to the C33A cell line from HPV-negative cervical cancer with the HcerEpic normal cervical epithelial cell line. Furthermore, CaSki cells having the highest HPV copy number demonstrated the highest expression of TMEM45A, and SiHa cells with the lowest HPV copy number had lower TMEM45A levels, suggesting that HPV copy number may have an effect on the expression of TMEM45A in cervical cancer cells. On the other hand, TMEM45A knockdown in SiHa and HeLa cells suppressed proliferation, arrested cells in S phase and promoted cell apoptosis, inhibited migration, invasion, and EMT, in addition to the expression of mesenchymal markers, vimentin and N-cadherin were downregulated conversely the epithelial marker E-cadherin was upregulated (29).

Another protein that favors tumor cells is the protein TMEM45B, recently it was shown that it is located in trans-Golgi, endosomes membrane and lysosome membrane (38). Also was predicted that TMEM45B to localize to ER, and plasma membrane, was predicted to have seven passes through the membrane and have properties to confer the protein thermal aggregation (13, 30, 91), TMEM45B is expressed in white adipose tissue of the epididymis (30). Recently, it has been related to a function in innate immunity (38), but its role in different types of cancer is already being studied. In lung cancer and pancreatic cancer, TMEM45B has been linked to oncogenic activity, and evidence suggests that this activity occurs through upregulation of the cell cycle and metastasis (32, 33). It has been proposed as a good candidate as a biomarker to differentiate metastatic melanoma from primary melanoma (35). It is also proposed as a biomarker of progression and metastasis in prostate cancer (36). Shen et al., reported that TMEM45B is upregulated in gastric cancer tissues and in the cancer cell lines BGC-823, MGC-803, SGC-7901, and HGC-27. A decrease in the expression of TMEM45B in HGC-27 cells reduced the capacity for cell proliferation, migration, and cell invasion, consequently increasing the expression of E-cadherin and decreasing the expression of the N-cadherin and vimentin. The JAK/STAT3 signaling pathway regulates the EMT. It is a constitutively active signaling pathway in gastric cancer, its expression was investigated in HGC-27 cell, and it was observed that the knockdown of TMEM45B significantly decreased phosphorylation and activation of the JAK2 and STAT3 proteins. These data suggest that the effect of TMEM45B overexpression on proliferation, migration, and invasion in gastric cancer cells could be mediated through the JAK/STA3 pathway (37).

Another example of TMEM45B overexpressed is in osteosarcoma cells. The silencing of TMEM45B in the U2OS cell line decreased cell proliferation, invasion, and migration and suppressed tumor growth *in vitro* and *in vivo* (34). The key signal of the Wnt/β-catenin pathway is β-catenin, which interacts with T-cell factor/lymphocyte enhancer (TCF/LEF) to regulate cell cycle and proliferation-related genes c-Myc and cyclin D1. The silencing of TMEM45B in the U2OS cell line reduced the expression of β-catenin, cyclin D1, and c-Myc, suggesting that TMEM45B favors the osteosarcoma progression through regulation of the Wnt signaling pathway (34). The Wnt signaling pathway is involved in embryonic development and in the self-renewal of tissues in adulthood (94, 96). However, in cancer, it has been widely recognized that it is aberrantly active due to alterations of the different proteins that participate in the regulation of this signaling pathway and favors the development of different types of cancer such as hepatocellular carcinoma, colorectal cancer, leukemias, breast cancer and gastric cancer (94, 97–99).

The TMEM119 gene encodes another TMEM protein in this section, also called OBIF (osteoblast induction factor) that encodes a protein that only one passes through the membrane (50, 100). Located in the cell membrane (41, 100), it may have localization in the ER and cytosol (39, 50). TMEM119 is involved in osteoclast differentiation, bone development and normal bone mineralization (100). TMEM119 is induced explicitly by parathyroid hormone, and through interaction with essential regulators in the BMP2 signaling pathway, such as Smad1/5 and Runx2, it promotes osteoblast differentiation (39). In addition, Tanaka et al. reported that TMEM119 seems necessary for BMP-2 to induce ATF4 levels, an essential transcription factor in osteoblast differentiation (40). It has also been reported that it could be a biomarker to differentiate resident microglial cells from blood-circulating macrophages in the brain (41).

On the other hand, in cancer overexpression of TMEM119 has been reported, specifically in osteosarcoma tissues, and has been associated with a poor prognosis. The knockdown of TMEM119 in the osteosarcoma cell lines U2OS and MG63 decreased cell proliferation by inhibiting the G1/S transition and also led to a decrease in the expression of Bcl2 and an increase in the expression of caspase 8 and caspase 9 most likely participating in the induction of apoptosis and suppressed tumorigenesis *in vivo* in nude mice and also decreased the ability of cell migration and invasion. Also the use of a TMEM119 siRNA led to a significant decrease in the metastatic factors MMP2, Twist1, ZEB1, vimentin, N-cadherin and α-SMA and an increase in an anti-metastatic factor E-cadherin. (42). A gene set enrichment analysis (GSEA) indicated that TMEM119 expression was strongly associated with the TGF-β pathway, which is involved in cell migration, and invasion (42). El knockdown of TMEM119 in the cell lines U2OS and MG63 TMEM119 decreased BMP2, BMP7, and TGF-β protein levels. While U2OS and MG63 cells transfected with TMEM119 siRNA were treated with TGF-β or vehicle, exposure to TGF-β rescued the effects of TMEM119 siRNA and significantly promoted osteosarcoma cell migration and invasion. In addition, in Saos2 cells that have a low expression of TMEM119, when overexpressing TMEM119 and treating them with a TGF-β inhibitor or with a BMP-type receptor inhibitor, a decrease in migration and invasion was observed, inverse effects to those caused by the due to the overexpression of TMEM119, these findings indicate that TMEM119 can promote cell migration and invasion by activating the TGF/BMP pathway (42).

Furthermore, a decrease in the expression of TMEM119 in gastric cancer cell line SGC-7901 reduced cell viability, favoring apoptosis (43). In another study from the same group, TMEM119 expression was reported to be increased in tumor gastric tissues compared to its non-tumor counterparts, and this overexpression was associated with a shorter lifespan (47). The low expression of TMEM119 produced by an interfering RNA in the SGC-7901 cell line significantly decreased cell invasion and migration (47). A correlation between STAT3 activation and the invasion, migration, and prognosis in gastric cancer was reported, as a positive correlation between STAT and VEGF expression was observed in this type of cancer (47). Therefore, it was evaluated whether the silencing of TMEM119 activates the STAT3 pathway and its downstream proteins MMP2 and MMP9. TMEM119 silencing reduced p-STAT3, MMP2, MMP9, and VEGF but had no effect on total STAT3 expression (47). On the other hand, the MKN45 cell line that overexpressed TMEM119 upregulated the expression of the p-STAT3, VEGF, MMP9, and MMP2 proteins but had no effect on total STAT3 expression. The use of a JAK2 kinase inhibitor significantly decreased migration and invasion induced by TMEM119 overexpression in the MKN45 cell line, which reduced the expression of p-STAT3, VEGF, MMP9, and MMP2 but did not affect STAT3 expression. These data together show that the oncogenic activity of TMEM119 in gastric cancer is through the activation of STAT3 signaling, although the exact mechanism of this activation is still unknown (47).

Recently, TMEM119 was reported to be overexpressed in ovarian cancer tissues, and expression levels are positively related to the International Federation of Gynecology and Obstetrics (FIGO) stages, suggesting that TMEM119 might be a prognostic biomarker. Knockdown of TMEM119 in A2780 and OVCAR-3 cell lines causes inhibition of cell proliferation, invasion, and migration through the PDGFRB/PI3K/AKT signaling pathway (44). Furthermore, from analysis of data sets available online, (45) reported that TMEM119 protein is overexpressed in breast cancer tissues compared to adjacent healthy tissue and that this overexpression predicts poor prognosis, suggesting that TMEM119 could be an oncogene in breast cancer. In the MDA-MB-231 cell line, overexpression of TMEM119 has been observed. In contrast, in the MCF-7 cell line, the expression is decreased, for which a knockdown in the MDA-MB-231 cell line and overexpression in MCF-7 were performed, and then, it was observed that the knockdown of TMEM119 reduces the expression of Sox2, Nanog, and OCT4 pluripotency markers. It also decreases the formation of spheroids, ALDH activity, migration, and invasion. In contrast, TMEM119 overexpression in the MCF-7 cell line favored all events that knockdown reduced. In addition, the EMT process was favored by the overexpression of TMEM119, while in the knockdown cells, this process was inhibited, demonstrated by the expression level of the EMT markers vimentin and E-cadherin, these data reveal that TMEM119 promotes pluripotency in cells of breast cancer. In addition, TMEM119 can activate the Wnt/β-catenin pathway in breast cancer cells (45). It was shown that β-catenin and TMEM119 have positive feedback as β-catenin has 3 binding sites to the TMEM119 promoter, upregulating the expression of TMEM119 in breast cancer cells, and in consequence, TMEM119 promotes pluripotency of breast cancer cells in a β-catenin dependent manner (45).

TMEM48, also called NCD1, is a protein conserved in eukaryotic cells in its C-terminal domain. It was localized to the nuclear membrane and was proposed as a nucleoporin required for the assembly of the nuclear pore complex (20). Six passes through the nuclear membrane have been described for this protein, and its N and C-terminal domains are cytoplasmic (101). TMEM48 is overexpressed in non-small cell lung cancer (NSCLC). TMEM48 mRNA levels were higher in 60 NSCLC tissues compared to non-tumor tissues. They were associated with a higher probability of developing advanced cancer, presenting lymph node metastases, larger tumors, and a shorter lifespan. Knockdown of TMEM48 with a shRNA in A549 and H1299 cells decreased cell proliferation and the percentage of cells in the G2/M phase, significantly increasing the rate percentage of cells in G0/G1 phase. Likewise, a decrease in the expression of genes involved in cell cycle progression was observed: cyclin B1, CDK1, and CDC6. This suggests that the silencing of TMEM48 suppresses the cell cycle transition. In addition, it reduced the expression of the PCNA and RFC4 genes involved in DNA replication (48). On the other hand, the decrease in the expression of TMEM48 impacted the increase of cell apoptosis, the expression of the pro-apoptotic gene Bax. It decreased the expression of the anti-apoptotic genes Bcl-2, XIAP, and survivin. Similarly, adherent ability, migration, and invasion in cell lines and *in vivo* tumorigenicity in mice were reduced; all these data together suggest a prognostic value for TMEM48 in NSCLC (48).

In a similar study, high levels of mRNA and TMEM48 protein expression were reported in 42 cervical cancer (CC) tissues compared to their adjacent healthy tissues; these data were consistent in the SiHa and HeLa cell lines. The use of an shRNA for TMEM48 in the SiHa and HeLa cell lines showed similar results to those observed by (48) previously described. The knockdown effect of TMEM48 was evaluated in the HeLa cell line and in a xenograft model in nude mice, keeping a downregulation of β-catenin expression and its target genes TCF1 and AXIN2. Since the Wnt/β-catenin signaling pathway is actively involved in the development of CC and other types of cancer, the functional association between TMEM48 and the Wnt/β-catenin pathway was evaluated using LiCl as a Wnt pathway activator in HeLa cells and proliferation and migration capacity were partially recovered. These data together suggest that TMEM48 has an oncogenic function in CC. The activation of the Wnt/β-catenin pathway could mediate so that TMEM48 could be a candidate as a prognostic biomarker or therapeutic target (49).

The transmembrane protein TMEM168, which localizes to the nuclear membrane, is predicted to have eleven passages across the membrane (50, 52). Its role in cell physiology is related to the maintenance of electrical stability through the regulation of the SCN5A protein on the cell surface (52). Its expression and function have been investigated in glioblastoma multiforme (GBM). Xu et al., detected high levels of TMEM168 mRNA in 85 GBM tissues compared to healthy tissues. These results were similar to those obtained from a glioblastoma dataset in The Cancer Genome Atlas (TCGA) program. The increase in TMEM168 expression was correlated with a shorter survival time. Downregulation of TMEM168 expression with a siTMEM168 in U87 and U373 cells, reduced cell proliferation by arresting the G0/G1 phase, increased cell apoptosis, and led to dysregulation of PCNA, survivin, cyclin D1, and Bax gene expression. At the same time, siTMEM168 was shown to inhibit the activation of the Wnt/β-catenin pathway and to reduce the expression of its target proteins c-Myc, cyclin D1, and survivin. Using LiCl in the U87 cell line, in which it is well known that the Wnt/β-catenin pathway modulates proliferation, the cell cycle, and apoptosis, the effect caused by siRNA on cell proliferation was opposite, confirming that the oncogenic effect of TMEM168, is through the Wnt/β-catenin signaling pathway (51).

In addition, the TMEM98 protein, encoded on chromosome 17, is a type II protein, and located in the cell membrane, exosome, also secreted (53, 55), and predicted located in ER (50). Exosomes can also secrete TMEM98, thus participating in T helper 1 (Th1) cell differentiation *in vitro* and *in vivo* (53). Recently, TMEM98 was reported to be involved in increased cell proliferation and migration in head and neck squamous cell carcinoma (HNSCC) (56). A bioinformatics analysis showed that the TMEM98 mRNA, in its 3´ UTR region has a binding site for Mir-29c-5p, at the same time an bioinformatics analysis showed that low levels of Mir-29c-5p expression were associated with a poor prognosis in patients with this type of cancer (56). In the FaDu and HSC-3 cell lines, the levels of Mir-29c-5p were lower concerning the INOE cell line, which was used as a control. Consistent with these data, the TMEM98 mRNA levels were higher in tissues with low Mir-29c-5p expression. With a Mir-29c-5p mimic, the expression of Mir-29c-5p was increased in the FaDu and HSC-3 cell lines, and cell proliferation and migration were reduced. While TMEM98 expression was decreased in the FaDu and HSC-3 cell lines, reducing cell proliferation and migration. Increased expression of Mir-29c-5p *in vivo* in nu/nu mice resulted in decreased tumor growth. These data suggest that TMEM98 has an oncogenic function in HNSCC, which is down-regulated by MiR-29c-5p (56).

TMEM98 has also been reported to be involved in increased cell proliferation in gastric cancer. The oncogenic role of TMEM98 has been shown to bat the mRNA level and not through the mature protein. Ao et al., analyzed 61 tissues from patients with gastric cancer and four gastric cancer cell lines MGC-803, BGC-823, SGC-7901, and MKN-45. The mRNA levels were higher than in healthy tissues, and GES-1 and RGM-1 cells from normal gastric mucosa. Furthermore, higher protein levels were reported in tumor cell lines relative to control cell lines. In the MKN-45 and SGC-790 cell lines, the expression of TMEM98 was reduced by a siRNA, and this led to a significant decrease in cell proliferation and invasion, in a dose-dependent manner of the siRNA. The overexpression of TMEM98 with a recombinant protein in MKN-45 and SGC-7901 cells showed that the effect on cell proliferation and invasion is mediated by the TMEM98 mRNA and not by mature protein since protein levels increased, however, without effect on cell viability and invasion (54). Computer analysis identified the TMEM98 mRNA target protein as the RNA-binding protein nuclear factor 90 (NF90) to which TMEM98 mRNA binds through an 8-nucleotide motif, but the underlying mechanism of this binding remains unclear, but, whether the cancer-promoting effect through TMEM98 mRNA (54).

## TMEM proteins also decrease cell proliferation, migration, and invasion in cancer

In counterpart, TMEM proteins have been related to an oncogenic activity that increases proliferation and migration in cell invasion through distinct signaling pathways, other TMEMs have a suppressive activity of tumors by negatively regulating proliferation, migration, and cell invasion.

TMEM106A, encoded on chromosome 17, it is located in the plasma membrane and in the mitochondria, contains only one passage through the membrane and is a type II membrane protein that lacks an N-terminal signal peptide (17, 50, 59). It belongs to the TMEM106 family, comprising the TMEM106A, TMEM106B, and TMEM106C proteins. The TMEM106A is conserved in humans, chimpanzees, monkey, dog, mouse, and rat. TMEM106A are involved in M1-type macrophage polarization through the MAPK and NF-κB signaling pathways (58). In some types of cancer, it has been associated with a tumor suppressor function (17, 59, 60). TMEM106A expression was found to be decreased in tumor tissue samples, and gastric cancer cell lines by hypermethylation of the promoter region. This low expression was associated with increased cell proliferation and in contrast, induced overexpression of TMEM106A in gastric cancer cell lines is related to decreased cell proliferation and increased apoptosis due to the activation of the caspase cascade (17). In renal cell carcinoma (RCC), reduced levels of mRNA and TMEM106A protein were reported in five cell lines RCC 786-O, 769-O, ACHN, A498, and A794 compared with the control cell lines of renal proximal tubular epithelium and primary renal cortical epithelium. In the ACHN and 786-O cell lines that presented lower expression of TMEM106A at baseline, TMEM106A was overexpressed, and this was achieved by decreasing the growth rate and cell migration capacity and adding the percentage of apoptotic and necrotic cells concerning the corresponding cell control (59). On the other hand, the decrease of the expression of TMEM106A with a siRNA in cell lines with relatively high levels of TMEM106A conferred the ability of colony formation and migration. These observations propose a tumor suppressor role for TMEM106A. TMEM106A activity in RCC is likely related to lower JNK kinase activation since cell lines that overexpress TMEM106A increased phosphorylation of the JNK protein. When the expression of TMEM106a diminished, phosphorylation of JNK also decreased (59).

In a similar study, it was reported that in NSCLC tissues, TMEM106A mRNA levels were reduced compared with adjacent normal lung tissues, and in A549, H1299, H1650, and H460 cell lines, mRNA accumulation and protein expression of TMEM106A was significantly lower compared to the control cell line. Cell lines A549 and H1299 showed the lowest levels of TMEM106A mRNA and protein; they were selected to overexpress TMEM106A, and it was observed that cell proliferation was significantly reduced, cell apoptosis, Bax, and caspase 3 expression were detected to increase, while Bcl-2 expression decreased. Furthermore, overexpression of TMEM106A reduced the expression of the mesenchymal markers N-cadherin and vimentin and increased the expression of E-cadherin, suggesting that TMEM106A reduced transition epithelial-mesenchymal. Because the PI3K/AKT and NF-kB signaling pathways play roles in the progression of different tumors, in apoptosis, migration, and cell invasion, it was investigated whether TMEM106A exerts an effect on these proteins and found that high levels of TMEM106A decreased the phosphorylation of PI3K, AKT, and P65 (NFκ-B), suggesting that TMEM106A has an inhibitory effect on activation of the PI3K/AKT/NF-κB pathway, however, the precise mechanism is still unknown (60).

The TMEM170B protein is encoded in chromosome 6 and was predicted to contain three transmembrane domains (50), it is located in the plasma membrane and in the cytoplasm (61, 62), is highly conserved from vertebrates to mammals (61). TMEM170B protein has been recognized as a negative regulator of the Wnt/β-catenin protumorigenic pathway in breast cancer (61). It was reported that the miR-27a downregulates TMEM170B in breast cancer and suggested a suppressor tumor role for TMEM170B. This is supported because the knockdown of TMEM170B in MCF7 cell line significantly induced the cell proliferation and colony formation. Furthermore, the overexpression of TMEM170B in the MDA-MB-231 cell line significantly impeded the breast cancer cells proliferation and colony formation. Likewise, the expression of TMEM170B was inversely correlated with the levels of β-catenin. When evaluating the expression levels of the target genes of the Wnt pathway, it was observed that the deletion of TMEM170B induced the protein levels of TCF4, CD44, c-Myc and cyclin D1 in MCF7 cells. In contrast, the overexpression of TMEM170B in cells MDA-MB-231 reduced protein levels of TCF4, CD44, c-Myc, and cyclin D. Through the analysis of the nuclear and cytoplasmic extracts, it can be corroborated that, the effect of TMEM170B in breast cancer is mediated by inhibition of the stabilization and translocation to the nucleus of β-catenin, It was shown that TMEM170B can directly regulate β-catenin expression independently of GSK-3β (61).

Additionally, TMEM170B was found to be significantly downregulated in pancreatic adenocarcinoma (PAAD), breast cancer, ovarian cancer, and thyroid cancer (62). In PAAD, the decreased expression of TMEM170B is associated with a poor prognosis. It is suggested that TMEM170B has a tumor suppressor role in PAAD related to inhibiting mutations in the TP53 gene. Likewise, its tumor suppressor effects are associated with the reduction of myeloid-derived suppressor cells and regulatory T cells and the infiltration of antitumor immune cells: CD8+T cells, CD4+T cells, and M1 macrophage in the tumor microenvironment (62).

**Figure 3:**
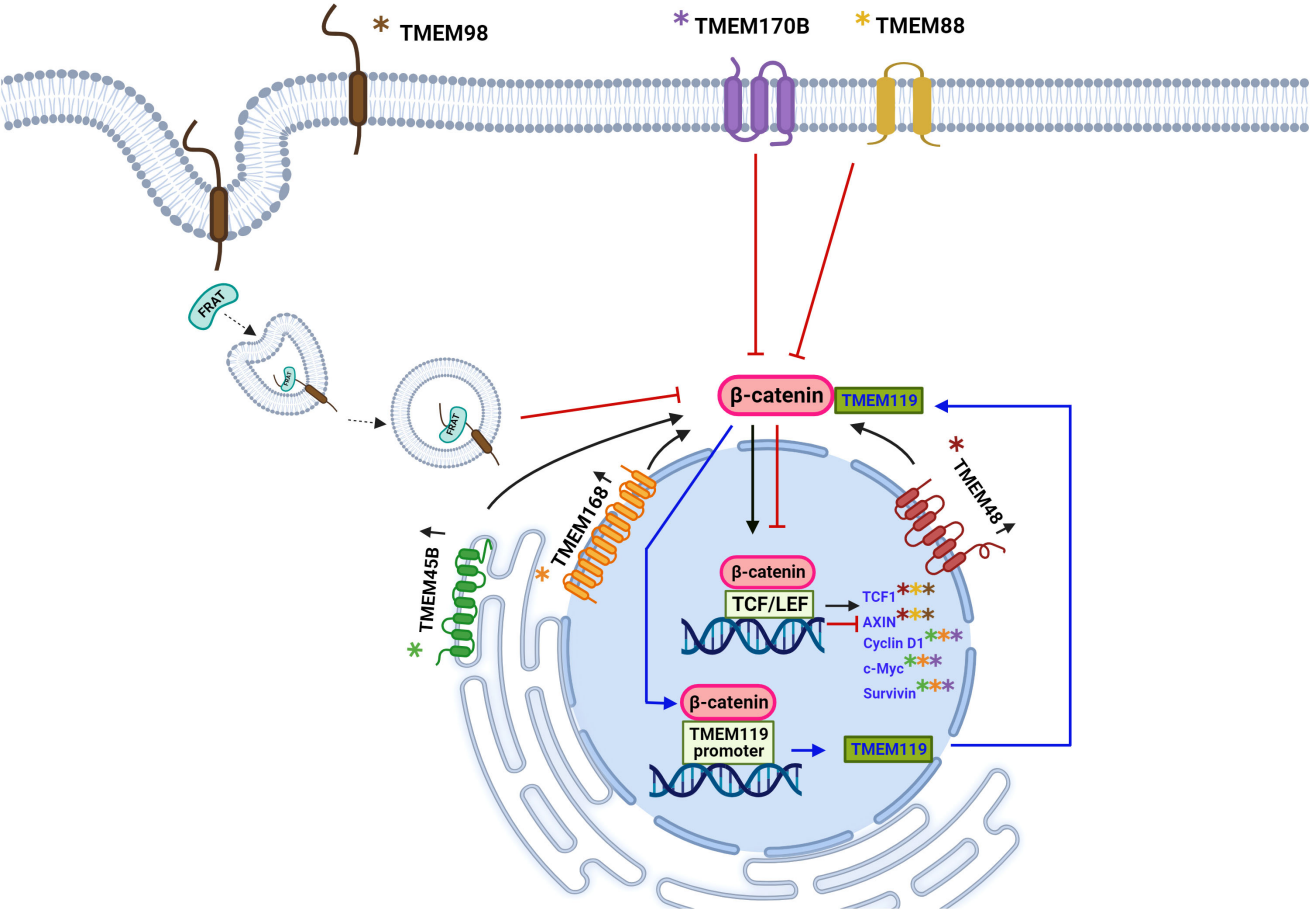
Schematic representation of the involvement of TMEM protein as oncogenes or tumor suppressors through participation in the Wnt signaling pathway. The Wnt signaling pathway has been aberrantly implicated in different types of cancer. The β-catenin protein is a key downstream component of the canonical Wnt signaling pathway. In the absence of the Wnt ligand, β-catenin forms a complex with AXIN1, AXIN2, APC, CSNK1A1, and GSK3B that promote phosphorylation at the N-terminal Ser and Thr residues and the ubiquitination of β-catenin and its subsequent degradation by the proteasome. Some TMEM proteins have been involved in signaling mediated by β-catenin, thus exerting an oncogenic force or tumor suppressors. For the TMEM119 protein, an oncogenic role has been described through positive feedback with β-catenin, three binding sites of β-catenina to the TMEM119 promoter were identified, which leads to the expression of the TMEM119 gene and, in turn, the TMEM119 protein was shown to it interacts with β-catenin and stabilizes it leading to the expression of its target genes. The overexpression of the proteins TMEM45B, TMEM48, TMEM168 is associated with an oncogenic role by favoring the translocation of β-catenin to the nucleus and the expression of its target genes: TCF1, AXIN, Cyclin D1, c-Myc and survivin, there is still no it is not clear by what mechanism these proteins induce the translocation of β-catenin. The TMEM170B and TMEM88 proteins are associated with a tumor suppressor role by decreasing or preventing the translocation of β-catenin to the nucleus. TMEM98 is associated with a decrease in β-catenin activity through its binding to the FRAT2 protein that positively modulates Wnt/β-catenin signaling. Created with Biorender.com.

So far, we are observed that some TMEM proteins, such as TMEM48, TMEM45B, and TMEM168, are involved in tumor growth, proliferation, migration, and cell invasion through the Wnt/β-catenin pathway, while TMEM170B and TMEM98 act as cancer developmental inhibitors through the same pathway. The exact mechanism of how TMEM proteins interact with the Wnt pathway is somewhat straightforward for some TMEM, while for other TMEM is entirely unknown. However, it is clear to us that transmembrane proteins play a role in cancer; either as oncogenic or tumor suppressor function is related to this signaling pathway Wnt (**Figures 2**, **3**).

## TMEM proteins with a dual function in different types of cancer

Emerging research has reported that the same TMEM protein may be involved in a tumor suppressor or oncogenic role depending on the type of cancer; here, we show these TMEM proteins.

The TMEM158 protein, encoded by the gene located on chromosome 3, is also known as RIS1, 40BBP, BBP, and HBBP tumor suppressor (71). The first reports on TMEM158 showed its function as a receptor for the neurotrophic peptide related to brain injury, which is crucial for the survival of neurons. Likewise, TMEM158 was upregulated during Ras-induced senescence in Ras-infected fibroblasts (70).

TMEM158 expression was significantly lower in prostate cancer tissues and is associated with disease progression and unfavorable clinicopathologic features such as advanced stage, lymph node invasion, residual tumors after surgery, elevated PSA, higher Gleason scores, lower disease-specific survival, older age, more aggressiveness of the disease. Likewise, the expression of TMEM158 was significantly lower in highly metastatic prostate cancer cells than in cells with less metastatic capacity. In addition, patients with higher TMEM158 expression had especially favorable overall survival outcomes and longer progression-free intervals. Likewise, it was reported that the expression of TMEM158 had a very negative correlation with the expression of the androgen receptor (AR), suggesting that the AR pathway negatively modulates the expression of TMEM158 in prostate cancers. Additionally, it was found that in prostate cancer, R-Ras signaling could be involved in the modulation of TMEM158 expression and be associated with the infiltration of natural killer cells and mast cells, playing an antitumor role (68)

On the other hand, the expression of TMEM158 has been reported to be upregulated in multiple types of cancer and has been associated with an oncogenic function (63–67, 69, 70). In laryngeal cancer, TMEM158 expression increased according to increasing TNM stage; patients with low TMEM158 expression had a significantly longer survival time than patients with high TMEM158 expression. TMEM158 knockdown inhibited LSC-1 cell viability, induced early apoptosis, and activated apoptotic factors Bax (67). Furthermore, through bioinformatic analysis, it was identified that in laryngeal cancer, TMEM158 is regulated by miR-548ac, and miR-548ac overexpression decreased TMEM158 levels. However, miR-548ac levels are significantly lower in tissues with carcinoma of the larynx compared to adjacent normal tissues, a higher expression of TMEM158 is observed and acts as an oncogene (67).

TMEM158 has been associated with an oncogenic role in ovarian cancer, increased cell proliferation, invasion, and adhesion by regulating the cell cycle cellular and proteins Transforming Growth Factor-β1 (TGFβ1), bone morphogenetic protein 4 (BMP4), adhesion molecule1 (ICAM1) and vascular cell adhesion molecule1 (VCAM1) (64). Additionally, in pancreatic cancer, it has been reported that TMEM158 is significantly overexpressed and is associated with increased tumor size and poor prognosis. In addition, TMEM158 has an oncogenic role by increasing the expression of TGFβ1, vimentin, N-cadherin, and α-SMA that induce the EMT process and PI3K/AKT signaling that also regulates cancer cell proliferation, metastasis in pancreatic cancer (65).

In colorectal cancer, TMEM158 overexpression promotes cell proliferation and migration and inhibits apoptosis. The knockdown of TMEM158 reduced tumor growth *in vivo* in a nude mouse (66). In triple-negative breast cancer, the lower expression of TMEM158 was associated with better overall survival and disease-free survival in patients. The knockdown of TMEM158 in BT49 and HCC1187 cell lines significantly decreased cell migration and invasion. In addition, the MDA-MB23 cell line that showed lower expression for TMEM158 was transfected to induce overexpressing TMEM158 the migration of an invasion cell was significantly increased, same as the cell proliferation and colony formation. The knockdown of the TMEM158 in HCC1187 and BT549 cells line downregulated the expression of N-cadherin and vimentin, also upregulating the expression of E-cadherin and altered the morphology cells conferring epithelial-like features. In contrast, overexpression TMEM158 in MDA-MB-231 had the opposite effects cells also changed their morphology, showing many fibroblast-like features. Knockdown of TMEM158 in HCC1187 and BT549 cells markedly reduce the expression of TGF-β1, -smad2/3, and p-ERK. Downregulation of the ERK1/2 signaling pathway with an inhibitor in MDA MB 231 cells led to inhibition of the TGF pathway. These results suggest that TMEM158 is involved in regulating tumor invasion and metastasis by EMT, activating both canonical and non-canonical TGF signaling pathways (70).

Emerging research has reported that in glioma, high levels of expression of TMEM158 are associated with a poor prognosis and shorter survival time in patients, and its expression level is higher in the more advanced stages of glioblastoma. Besides, overexpression of TMEM158 in cell lines U87MG, U251MG, and TJ905 increased proliferation, migration, invasion, and colony formation efficiency, while downregulation also decreased these processes. In addition, TMEM158 was overexpressed in U87MG, U251MG, and TJ905 glioma cells, E-Cadherin was downregulated while N-Cadherin, vimentin, and Snail was detected. Likewise, the negative regulation of TMEM158 in glioma cells significantly increased the expression of E-Cadherin and decreased the expression of N-Cadherin, vimentin, and Snail. TMEM158 can potentially enhance glioma cell motility by activating the EMT process. Furthermore, TMEM158 positively correlates with STAT3 signaling in glioma cells, and regulates the glioma cell proliferation, migration, invasion, and EMT process by activating STAT3 signaling (69).

A recent pan-cancer study where TMEM158 expression levels were analyzed in 33 types of cancer using the TCGA and Genotype-Tissue Expression (GTEx) databases suggests that TMEM158 may have distinct biological functions in various cancer tissues. TMEM158 overexpression in adrenocortical carcinoma, bladder urothelial carcinoma, cervical cancer, kidney renal clear cell carcinoma, kidney renal papillary cell carcinoma, brain lower grade glioma, lung adenocarcinoma, mesothelioma and PAAD was associated with a poor prognosis. On the other hand, in over 20 types of cancer TMEM158 is associated with cancer-associated fibroblast infiltration. The study reports that the immune function of TMEM158 is linked to the co-expression of two immune checkpoint genes, CTLA4 and LAG3. Gene pool enrichment analysis of the GSA gene pool demonstrated that TMEM158 plays an essential role in the cancer immune response and could be considered a potential therapeutic target to modulate the immune system in cancer patients. Furthermore, there is evidence that TMEM158 may be involved in chemotherapeutic sensitivity and cancer progression by activating signaling pathways such as TGF1 and PI3K/AKT. Therefore, TMEM158 becomes a promising therapeutic target or prognostic biomarker that should continue to be studied until its role is fully understood in the different types of cancer where it has been differentially expressed (71).

The TMEM88 protein is encoded on human chromosome 17, is located in the plasma membrane, and has been identified with cytosolic localization (50, 81, 82); the function of TMEM88 has been characterized in Xenopus embryos where it interacts with the PDZ domain (Post synaptic density-95, disc large and zonular occludes-1) of the Disheveled one protein (Dvl 1/Dsh), a key element of the Wnt signaling pathway, this interaction occurs through the VWV motif (Val-Trp-Val) located in the C-terminal region of TMEM88 that competes with LRP5/6, leading to Dvl being recruited into the plasma membrane and inhibiting the canonical Wnt pathway (77). In humans, two isoforms have been reported: TMEM88 CRA_a and TMEM88 CRA_b;only the CRA_a isoform has the PDZ binding VWV domain that allows it to regulate the Wnt/β-catenin pathway (79). In humans, TMEM88 is involved in cardiomyocyte differentiation and heart development by downregulating the Wnt/β-catenin pathway (79). In cancer, TMEM88 has been associated with a tumor suppressor and oncogenic function determined by its cellular location (25, 72). It was reported that in NSCLC, they identified the expression of TMEM88 in the plasma membrane and in the cytosol. Membrane-associated overexpression of TMEM88 in A549, H1299, H460, H292, SPC-A1, LTEP-A-2, LK2, PG-BE1, and PG-LH7 cell lines was found to inhibit the Wnt/β-catenin pathway, by reducing the expression of its target genes cyclin D1, c-Myc, and MMP7, consistently, a decreased proliferation, colony formation, migration, and invasion, as well as a decrease in tumor growth *in vivo* was observed. These findings show that TMEM88 has a tumor suppressor function when associated with the membrane by inhibiting the Wnt/β-catenin pathway (25).

A similar study reported low levels of mRNA and TMEM88 protein in thyroid cancer tissues compared to adjacent healthy tissues, as well as in thyroid cancer cell lines BCPAP, TPC1, K1, and NPA87 relative to the control cell line Nthy- Ori-3-1. It was demonstrated that the overexpression of TMEM88 conferred to the cells the ability to inhibit growth *in vitro* and *in vivo*, in addition to the fact that cell lines that overexpressed TMEM88 also reduced cell invasion and colony formation, while the decrease in the expression of TMEM88 with an shRNA favored cell growth and invasion, suggesting a tumor suppressor activity in thyroid cancer. At the same time, this tumor suppressor activity of TMEM88 was shown to be related to its downregulation that it exerts on the Wnt/β-catenin signaling pathway since the decrease in TMEM88 increased the expression of the active form of β-catenin and the transcriptional activity of TCF/LEF and in turn, the expression of its target genes c-Myc, and cyclin-D1, for which, TMEM88 could be studied for its use as an antitumor target (75).

A recent investigation reported that the TMEM88 protein is downregulated in hepatocellular carcinoma (HCC) tissues and that high levels of TMEM88 predict better overall survival. When the TMEM88 protein was overexpressed in Huh7 cells, a decrease in cell proliferation capacity was observed. Furthermore, it was followed by *in vivo* analysis using nude mice that overexpression of TMEM88 can markedly suppress HCC progression. Therefore, TMEM88 could be a prognostic factor in HCC; for this, it is necessary to continue studying this protein to identify the mechanism through which it exerts a suppressive effect on tumors in HCC (73).

TMEM180 protein is found in the plasma membrane, 12 transmembrane domains were identified, and its N- and C-terminal domains are located in the extracellular space; it is proposed that it could act as a cation importer (11). TMEM180 has been proposed to have an essential in glutamine and arginine uptake or metabolism in SW480 colorectal cancer cells (83). Subsequently, TMEM180 was identified as contributing to the growth of SW480 cells by altering metabolism and promoting the expression of enzymes in the nitric oxide (NO) synthesis system, suggesting that it promotes glucose and glutamine metabolism, and this in turn, to tumor growth (84). In addition, in colorectal carcinoma cells, a decreased expression suppressed cell proliferation (11, 84). High expression of TMEM180 is associated with a shorter lifespan in a patient with stage III colorectal cancer; it was also reported that *in vitro* and *in vivo* in mice, the SW480 colorectal cancer cell line showed tumor initiation ability, and this was positively associated with the level expression of TMEM180 so in colorectal cancer TM180 acts as an oncogene (85). On the other hand, Mei et al., Reported that in pancreatic cancer cells, the decrease in the expression of TMEM180 with a siRNA favored cell proliferation. Consequently, it could have a role as a tumor suppressor in pancreatic cancer (92).

On the other hand, the transmembrane protein TMEM98, which we previously mentioned an oncogenic role that has been shown in some types of cancer; it could also exert a suppressive role in tumors. Some reports suggested that TMEM98 binds to the FRAT2 protein and inhibits its positive modulation activity of the Wnt/β-catenin pathway by mediating the binding of FRAT2 to the GSK3 protein (55). This opens the possibility that TMEM98 acts as a tumor suppressor at the protein level. Recently, it was reported that TMEM98 exerts a tumor-suppressor effect on ovarian cancer (57). The low expression of TMEM98 in ovarian cancer is correlated with shorter survival time in patients, promoted proliferation, migration, invasion, vasculogenic mimicry, and inhibited apoptosis in SKOV3 and IOSE80 ovarian cancer cell lines. In addition, low expression of TMEM98 promoted ovarian cancer development *in vivo* in female nude mice (57).

## TMEM proteins and their role in the response to chemotherapy

Resistance to drugs for cancer treatments is one of the main problems faced by patients with a neoplasm. Numerous proteins interfere at different levels in tumors to prevent the cytotoxic effect of drugs avoiding the entry of the drug into the cell or its arrival at the target site (102). However, the exact mechanisms remain largely unknown, and further investigation of those mechanisms and the identification of proteins involved is necessary to design more effective and efficient therapies.

It has been found that some TMEM proteins are involved in resistance to different antineoplastic drugs, such as the case of gene TMEM45A, which is positively regulated under hypoxic conditions by the hypoxia -inducible factor (HIF1α) (89). Hypoxia favors an oncogenic role for TMEM45A in breast cancer cells and hepatocellular carcinoma cells, favors the chemoresistance to paclitaxel and etoposide through resistance to apoptosis in response to hypoxia in breast cancer MDA-MB-231 cells and hepatocellular carcinoma HepG2 cells respectively (21).

In HNSCC and RCC, an increased TMEM45A mRNA accumulation was detected in tumor tissues compared to healthy tissues of each organ. However, it was observed that the response to treatment with cisplatin in the cell lines SQD9 from HNSCC and RCC4 + pVHL from RCC was different. In SQD9 cells, the knockdown of TMEM45A increased the sensitivity of the cells to cisplatin. This was mainly due to the silencing of TMEM45A, which affected the repair of DNA damage caused by cisplatin through a lower phosphatase activity of the EYA3 protein, which acted on γH2AX and decreased the recruitment of proteins involved in DNA damage repair, such as RAD51 recombinase, this could be the reason that led to cell death. On the other hand, in the RCC4 + pVHL cell line, resistance to cisplatin is increased due to the activation of the UPR pathway, which is involved in post-translational protein modification. In contrast, in SQD9 cells, the activation of this pathway was lower and the response to cisplatin was higher. These results show that TMEM45A could be a therapeutic target in cancer with a different approach depending on each type of cancer (26).

In colorectal cancer, TMEM45A is associated with multidrug resistance (MDR). In cell lines HCT-8/5-FU and SW480/5-FU resistant to 5-fluorouracil (5-Fu), a chemotherapeutic used in the treatment of colorectal cancer, the protein and mRNA levels of TMEM45A were significantly elevated concerning parental cells HCT-8/5 and SW480 (28). Furthermore, the knockdown of TMEM45A in HCT-8/5-FU and SW480/5-FU cells decreased MDR and increased the cell sensitivity to 5-Fu. These data suggest that TMEM45A might be involved in MDR in colorectal carcinoma. On the other hand, MDR increased the expression of TGF-βr1 and enhanced the phosphorylation of SMAD2/3 in HCT-8/5-FY and SW480/5-FU cells. These data suggest that these events could be regulated by the pathway TGF-β signaling in colorectal cancer cells (28).

In addition to the previously mentioned effects of TMEM45A on CC cell lines, the impact of TMEM45A on the response to cisplatin was also evaluated in cisplatin-resistant SiHa/DDP and HeLa/DDP cells and reported a higher expression of TMEM45A than in their counterparts SiHa and HeLa parent cells. TMEM45A knockdown reduced the cisplatin resistance of HPV-positive CC cells. Furthermore, TMEM45A was upregulated in SiHa/DDP and HeLa/DDP cells relative to their parental cells, respectively. TMEM45A knockdown reversed the cisplatin resistance of HPV-positive cervical cancer cells by increasing apoptosis (29).

TMEM158, it has also promoted cisplatin chemoresistance in lung cancer cells. Knockdown of TMEM158 in cisplatin-resistant NSCLC PC-9/CDDP cell lines increased the sensitivity of the cells to cisplatin. TMEM158 can function as a potent predictive biomarker for CDDP therapy in NSCLC (63). The TMEM158 protein, in addition to favoring other cellular processes previously mentioned in colorectal cancer (66). Also, TMEM158 expression levels are associated with multidrug resistance to cisplatin and 5-fluorouracil-resistant HCT116 and SW480 cell lines. TMEM158 influenced the expression of MDR related proteins such as MDR1, MRP1, and Bcl-2 when HCT-116 and SW480 cells were transfected with LV-TMEM158-GFP to overexpress TMEM158. In addition, when the expression levels of TMEM158 were decreased using a siRNA transfected in HCT116 and SW480 cells, the expression levels of MDR1, MRP1, and Bcl-2 proteins were significantly reduced (66). Therefore, TMEM158 is a good candidate as a biomarker of treatment response and therapeutic target.

## Conclusions

Physiological functions have been identified for many of the proteins classified in the TMEM family, roles that are entirely relevant to healthy cells. However, many of these proteins also have altered expression in different neoplasms. TMEM proteins play regulatory roles in the tumorigenesis and progression of various tumors through alterations in the capacity for cell proliferation, migration, invasion, EMT, and response chemotherapeutic, which are processes regulated at different levels, and the evidence showed that other proteins of the TMEM family significantly favor these processes. Also, some TMEM proteins act as tumor suppressor proteins depending on the type of cancer.

The question of whether TMEMs act as oncogenes or tumor suppressors and how they do motivate their study since they are considered potential candidate biomarkers for prognosis, diagnosis, response to treatment, or therapeutic targets and to understand what role each altered protein plays in cancer helps us to understand better to understand neoplasms better and thus lead to better treatments for patients in the future.

## Author contributions

SE-G contributed to the conception of the study. GH-Q contributed to the manuscript´s design and also designed and drew all figures and tables. All authors contributed to the article and approved the submitted version.
